# Perspectives of family medicine physicians on the importance of adolescent preventive care: a multivariate analysis

**DOI:** 10.1186/s12875-016-0402-6

**Published:** 2016-01-20

**Authors:** Jaime L. Taylor, Matthew C. Aalsma, Amy L. Gilbert, Devon J. Hensel, Vaughn I. Rickert

**Affiliations:** Indiana University School of Medicine, 410 W. 10th Street, Room 1001, Indianapolis, IN 46202 USA

**Keywords:** Adolescent, Preventive care, Primary care, Provider beliefs

## Abstract

**Background:**

The study objective was to identify commonalities amongst family medicine physicians who endorse annual adolescent visits.

**Methods:**

A nationally weighted representative on-line survey was used to explore pediatrician (*N* = 204) and family medicine physicians (*N* = 221) beliefs and behaviors surrounding adolescent wellness. Our primary outcome was endorsement that adolescents should receive annual preventive care visits.

**Results:**

Pediatricians were significantly more likely (*p* < .01) to endorse annual well visits. Among family medicine physicians, bivariate comparisons were conducted between those who endorsed an annual visit (*N* = 164) compared to those who did not (*N* = 57) with significant predictors combined into two multivariate logistic regression models.

Model 1 controlled for: patient race, proportion of 13-17 year olds in provider’s practice, discussion beliefs scale and discussion behaviors with parents scale. Model 2 controlled for the same first three variables as well as discussion behaviors with adolescents scale. Model 1 showed for each discussion beliefs scale topic selected, family medicine physicians had 1.14 increased odds of endorsing annual visits (*p* < .001) and had 1.11 greater odds of endorsing annual visits with each one-point increase in discussion behaviors with parents scale (p = .51). Model 2 showed for each discussion beliefs scale topic selected, family medicine physicians had 1.15 increased odds of also endorsing the importance of annual visits (*p* < .001).

**Conclusions:**

Family medicine physicians that endorse annual visits are significantly more likely to affirm they hold strong beliefs about topics that should be discussed during the annual exam. They also act on these beliefs by talking to parents of teens about these topics. This group appears to focus on quality of care in thought and deed.

## Background

Although most adolescents are considered physically healthy, adolescents still face significant morbidity and premature mortality [1]. Morbidity and mortality are often related to risk taking behaviors that include injury-related behaviors, violence, tobacco use, alcohol and drug use, unsafe sexual practices, inadequate physical activity and poor dietary habits [2]. Risk taking behaviors are frequently preventable, and for this reason, the American Academy of Pediatrics’ Bright Futures, the American Academy of Family Physicians (AAFP), the Society for Adolescent Health and Medicine (SAHM) and the American Medical Association (AMA) recommend annual preventive visits for adolescents [3–5].

Annual well visits, along with other primary care services, are most often provided to adolescents by pediatricians or family medicine physicians (FMPs) [6]. While both pediatricians and FMPs are adequately trained to care for adolescents, their training experiences vary significantly [6].

In 1997, the Accreditation Council for Graduate Medical Education (ACGME) mandated that pediatric residents complete a month-long adolescent medicine rotation because of the increasing complexity of adolescent health and well-being [7, 8]. While this requirement provides increased contact with adolescents, the experience and exposure to adolescent-specific topics varies across residency programs [7].

Family medicine residency programs, unlike pediatric residency programs, are not required by the ACGME to have a specific rotation in adolescent medicine. In fact, the ACGME is non-specific in their recommendations regarding adolescent medicine training in family medicine residency programs. [9] The AAFP, however, provides more detailed guidelines for family medicine residents surrounding adolescent health; specifying certain competencies, attitudes, knowledge and skills FMPs should obtain during residency in order to be prepared to manage adolescent health concerns in practice [10]. The AAFP recommends that this adolescent medicine curriculum be implemented via individual teaching, small group discussions, web-based resources, and didactics [10].

While adolescent-specific training for pediatricians and FMPs is significantly different, research suggests that discrepancies exist between the skills obtained from both types of training and the skills actually required for practice [11]. The purpose of this study was to examine differences between pediatricians and FMPs regarding their beliefs surrounding the importance of annual adolescent well visits in an effort to ascertain whether the type of training has an effect on this important recommendation and to examine how physician characteristics, beyond specialty, may impact beliefs regarding the importance of annual well visits for this population.

## Methods

The survey used for this study was designed to gather information regarding adolescent wellness and barriers to health care services. The online survey, known as the Perceptions of Adolescent Health (PATH) survey, was developed by Harris Interactive Incorporated with involvement from the National Foundation of Infectious Diseases (NFID) and Pfizer Inc. The survey had three distinct populations of focus: adolescents (13-17 years old), parents of adolescents and health care providers (HCPs). HCPs included physicians, nurse practitioners, physician assistants and licensed practical nurses in the fields of family medicine, general practice, internal medicine and pediatrics. For the purpose of this study, we used responses from providers who identified themselves as pediatricians or FMPs as these physician groups provide the majority of primary care to adolescents [6]. The nature of this study allowed for waiving of written informed consent which, along with study protocols, was approved by the Institutional Review Board at Indiana University-Purdue University, Indianapolis.

Survey data were obtained from December 2012 to January 2013. Survey respondents were required to meet certain criteria, i.e. time working directly in patient care, patients seen per month, and exposure to adolescent patients (13-17 years old). Subsequently, the data were weighted by Harris Interactive to be representative of the U.S. population as a whole. Descriptions of development and weighting have previously been described [12].

### Predictor variables

The survey collected data on provider demographics (gender, age, residency graduation year) as well as demographics of each provider’s patient population (patient race, patient age and patient reminders for well visits). Providers were asked about their familiarity with recommendations for adolescent health care and whether they believed well visits should occur annually or less frequently. The endorsement of annual adolescent well visits was the primary outcome for this study.

This online survey examined provider beliefs about the perceived importance of specified clinical discussions to the overall health of adolescent patients. Provider opinions regarding the level of knowledge held by adolescents about their own health as well as how proactive adolescents are in maintaining said health were also assessed. Lastly, the survey examined provider behaviors surrounding discussions with parents and adolescents regarding general health and wellness.

Provider age was subsequently collapsed into those less than fifty years old or greater than or equal to fifty years old. In addition, residency training completion was categorized into those who graduated prior to 2000 and those who graduated residency in or after 2000. This classification was based on the change in ACGME requirements for adolescent medicine specific training in Pediatrics.

Physicians reported on adolescent knowledge and proactivity separately using a 4-point likert scale ranging from “not at all knowledgeable/proactive” to “very knowledgeable/proactive”. For analyses, these variables were transformed into bivariate variables represented by “not at all to somewhat knowledgeable/proactive” and “knowledgeable/proactive to very knowledgeable/proactive”.

Physicians reported on how important they believed discussions about 11 topics were to the overall well-being of adolescent patients. These topics included weight, nutrition/diet, exercise, vaccines, issues at home, issues at school, self-image, sexual health, substance use, mental health and coping with stress, and again, importance was rated using a 4-point scale from “not at all important” to “very important.” Prior to analyses, responses were summed to create a Discussion Belief Scale (DBS). Scores ranged from 9 to 33 with a mean of 26.13 and a standard deviation of 5.80.

Provider discussion behaviors with adolescents and parents were assessed separately. Physicians were asked whether they regularly talk with adolescents and parents of adolescents about 12 topics including weight, nutrition/diet, exercise, vaccines, issues at home, issues at school, self-image, sexual health, substance use, mental health, prescriptions/medications and coping with stress. Responses were summed to create a Discussion Behaviors with Parents Scale (DBPS) and a Discussion Behaviors with Adolescents Scale (DBAS). Scores for the DBPS ranged from 0 to 12 with a mean of 5.95 and standard deviation of 3.38. Scores for the DBAS ranged from 0 to 12 as well with a mean of 9.68 and a standard deviation of 3.03.

### Outcome variables

Our primary outcome was physician endorsement that adolescent well visits should occur annually. Pediatricians were significantly more likely than FMPs (*p* < .01) to endorse annual visits. Accordingly, we focused our remaining analyses on FMPs to identify which factors were associated with the endorsement of annual visits by this subspecialty group.

### Analysis

Bivariate comparisons (using Chi-Square or Analysis of Variance) were conducted between those FMPs who endorsed an annual visit (*n* = 164) as compared to those who did not (*n* = 57). Significant predictors (*p* < .05) were combined into two multivariate logistic regression models.

We used these two multivariate logistic regression models to separately model the influence of the DBPS and the DBAS (on the endorsement of annual well visits). All models controlled for patient race, patient age (proportion of 13 to 17 years olds in the provider’s practice), and the DBS. All analyses were performed using SPSS, 22 and STATA, 12.0.

## Results

The initial sample consisted of 204 pediatricians and 221 FMPs. We computed a Chi-Square test to determine whether a significant difference existed between pediatricians and FMPs regarding the importance of annual adolescent well visits. Almost all pediatricians (*n* = 193) strongly endorsed the importance of annual well visits while less than three-quarters of FMPs agreed with this statement. (*p* < .001). As a result, the remaining analyses contrasted those FMPs who recognized the importance of annual adolescent well visits (*n* = 164) against those who did not (*n* = 57).

FMPs who believed well visits should occur annually compared to those who did not were similar in gender, age, and residency graduation year (Table 1). Their practices did not significantly differ based on standing policies for well visit reminders or race except for the proportion of Asian/Pacific Islanders in the practice. Those FMPs who endorsed annual adolescent well visits reported a slightly larger percentage of Asian/Pacific Islanders within their practice as compared to those who did not endorse annual well visits. Also, those who endorsed annual well visits had a significantly larger percentage of patients between the ages of 13 and 17 years old.

**Table 1:** Sample characteristics reported by Family Medicine Physicians based on how frequently they believe adolescent well visits should occur*

	Less than Annual Well Visits (%)	Annual Well Visits (%)	P value	
Provider Characteristics				
Gender of provider			.500	
Male	70.7	65.9		
Female	29.3	34.1		
Age of provider			.245	
< 50 years old	29.8	38.4		
≥ 50 years old	70.2	61.6		
Provider residency graduation (year)			.785	
Prior to 2000	80.7	82.3		
2000 or after	19.3	17.7		
Practice Demographics				
Patient race, mean (SD)				
White	61.43 (24.37)	60.37 (21.47)	.755	
Black	16.45 (20.68)	13.56 (1.06)	.536	
Hispanic	16.27 (17.15)	15.11 (1.18)	.883	
Asian, Pacific Islander	4.89 (5.13)	6.65 (.520)	.007	
Other	2.48 (.328)	1.25 (3.39)	.543	
Patient age, mean (SD)				
11-12 years old	5.03 (3.83)	5.64 (3.73)	.292	
13-17 years old	6.04 (3.81)	7.30 (3.94)	.037	
18-24 years old	11.23 (6.14)	11.16 (7.37)	.834	
Provider office has process for well visit reminders			.96	
No	49.1	36.6		
Yes	50.9	63.4		

*Percentages based on a nationally representative weighted sample

The two groups of FMPs did not significantly differ in regard to their familiarity with the annual well visit recommendation for adolescents or their beliefs regarding whether teens were knowledgeable about their overall health and whether the physicians believed that teens were proactive in efforts to maintain their health. However, those FMPs who endorsed the importance of annual visits were found to have significantly higher scores on the DBS than those who did not endorse annual adolescent visits with a mean of 27.45 and a standard deviation of 4.89 and a mean of 22.36 and a standard deviation of 6.54 (*p* < .01), respectively. Lastly, those FMPs who endorsed annual adolescent visits were significantly more likely to have higher scores on the DBPS and the DBAS than those who did not endorse annual visits with a mean of 6.46 and a standard deviation of 3.55 compared to a mean of 4.48 and a standard deviation of 2.30 (*p* < .01) on the DBPS and a mean of 10.01 with a standard deviation of 2.91 (*p* < .01) and a mean of 8.75 with a standard deviation of 3.18 (*p* < .01) on the DBAS.

Controlling for confounding variables, we identified two variables associated with FMPs who endorsed the importance of an adolescent annual visit (Table 2). Specifically, for each unit increase in their score on the DBS as well as each one unit increase in their score on the DBPS, there was a 1.14 and 1.11, respectively, greater odds of endorsing the importance of the adolescent annual visit. Our second model investigated confounding variables as well as scores on the DBAS, and we found that higher scores on the DBS were significantly associated with endorsing the importance of an annual adolescent visit.

**Table 2:** Family medicine physicians who endorse annual adolescent well visits

	Model 1: OR (95%CI)	Model 2: OR(95%CI)
Patient race (Asian/Pacific Islander)	1.07 (.990-1.15)^^^	1.08 (1.00-1.16)^^^
Patient age (13 to 17 years old)	1.07 (.901-1.28)	1.07 (.904-1.26)
Importance of preventative care discussion (Discussion belief scale)	1.14 (1.06-1.23)*	1.15 (1.07-1.24)*
Preventative care discussed with parents (Discussion behaviors with parents scale)	1.11 (1.00-1.24)^^^	-----
Preventative care discussed with adolescents (Discussion behaviors with adolescnets scale)	-----	1.05 (.890-1.24)

^^^p<.1, *p<.01

## Discussion

Almost all of the pediatricians surveyed recognized and endorsed the importance of annual well visits for adolescents, but only 3 of 4 FMPs had a similar belief. Practice and training differences between the fields may account for this significant disparity. Adolescent medicine-specific training among pediatricians and FMPs also varies. That is, pediatric residents complete an intensive one month rotation in adolescent medicine while family medicine residents receive their adolescent training over the course of three years in a semi-structured format. While the focus of care should be on the whole person and FMPs must continue to be trained broadly [13], our findings may demonstrate a need for improved education surrounding best practice guidelines. It may also be important to investigate FMPs’ knowledge of other adolescent-related guidelines and management of sensitive issues.

Our research shows that FMPs who endorse the importance of discussing an array of preventive health care topics, and then follow through with these broad discussions, are significantly more likely to also endorse the importance of annual well visits. Previous research suggests well visits are more accommodating to preventive counseling [14]. FMPs who endorse annual well visits may be are familiar with this evidence or may have personal experience that supports their belief that annual well visits are important for preventive care discussions. Moreover, these FMPs may be familiar with the causes of adolescent morbidity and early mortality and therefore recognize that discussing many topics at one visit could improve their ability to decrease high-risk behaviors. Possible differences in education, training, patient population in residency clinics or physician interest in specific patient populations could explain this relationship. More research is needed to understand why some FMPs utilize the annual well visit for the implementation of preventive health care discussions.

Our study suggests that those FMPs who endorse annual well visits are more likely to discuss prevention with the parents of adolescents, but not necessarily with the adolescents themselves. This interesting finding requires further research especially because previous research confirms that both parents [15, 16] and adolescents [16, 17] desire discussions surrounding preventive health care from their providers.

As with all research, there were limitations to this study. These data were, as mentioned, weighted by Harris Interactive to represent the U.S. population as a whole. It was not possible, however, to ascertain whether there was bias present during the participant selection because of our inability to track the number of providers initially asked to participate in the survey. Also, those FMPs who endorsed annual adolescent well visits reported a slightly larger percentage of Asian/Pacific Islanders within their practice. It is unclear why this difference existed. A limitation within the survey is the lack of information needed to investigate whether or not this represented a sampling bias. Because data was weighted to be nationally representative, this is unlikely. However, future research might focus on how the relationship between provider and patient race may impact the results found here.

## Conclusion

Primary care physicians, especially FMPs, have the unique ability to anticipate developmental issues, monitor behavior changes and intervene early with their patients as they often have a long lasting relationship and well-developed rapport. [16] Those FMPs who recognize the importance of annual well visits also endorse the importance of discussion surrounding preventive health care with this population. Further research is needed to investigate why there is a significant difference among FMPs and their endorsement of annual well visits as well as topics they believe to be important to discuss with both adolescents and their parents.

## Abbreviations

AAFP: American Academy of Family Physicians
ACGME: Accreditation Council for Graduate Medical Education
DBAS: Discussion Behaviors with Parents Scale
DBAS: Discussion Behaviors with Adolescents Scale
DBS: Discussion Belief Scale
FMPs: family medicine physicians
U.S.: United States
